# Exposure *in vitro* to an Environmentally Isolated Strain TC09 of *Cladosporium sphaerospermum* Triggers Plant Growth Promotion, Early Flowering, and Fruit Yield Increase

**DOI:** 10.3389/fpls.2018.01959

**Published:** 2019-02-01

**Authors:** Zhijian T. Li, Wojciech J. Janisiewicz, Zongrang Liu, Ann M. Callahan, Breyn E. Evans, Wayne M. Jurick, Chris Dardick

**Affiliations:** ^1^Appalachian Fruit Research Station, United States Department of Agriculture – Agricultural Research Service, Kearneysville, WV, United States; ^2^Food Quality Laboratory, Beltsville Agricultural Research Center, United States Department of Agriculture – Agricultural Research Service, Beltsville, MD, United States

**Keywords:** microbial volatile organic compounds, *Cladosporium sphaerospermum*, plant growth promotion, biostimulant, increased productivity, *Nicotiana tabacum* L., *Capsicum annuum* L., expression profiling

## Abstract

A growing number of bacteria and fungi have been found to promote plant growth through mutualistic interactions involving elements such as volatile organic compounds (VOCs). Here, we report the identification of an environmentally isolated strain of *Cladosporium sphaerospermum* (herein named TC09), that substantially enhances plant growth after exposure *in vitro* beyond what has previously been reported. When cultured on Murashige and Skoog (MS) medium under *in vitro* conditions, tobacco seedlings (*Nicotiana tabacum*) exposed to TC09 cultures for 20 days increased stem height and whole plant biomass up to 25- and 15-fold, respectively, over controls without exposure. TC09-mediated growth promotion required >5 g/L sucrose in the plant culture medium and was influenced by the duration of exposure ranging from one to 10 days, beyond which no differences were detected. When transplanted to soil under greenhouse conditions, TC09-exposed tobacco plants retained higher rates of growth. Comparative transcriptome analyses using tobacco seedlings exposed to TC09 for 10 days uncovered differentially expressed genes (DEGs) associated with diverse biological processes including cell expansion and cell cycle, photosynthesis, phytohormone homeostasis and defense responses. To test the potential efficacy of TC09-mediated growth promotion on agricultural productivity, pepper plants (*Capsicum annuum* L.) of two different varieties, Cayenne and Minisweet, were pre-exposed to TC09 and planted in the greenhouse to monitor growth, flowering, and fruit production. Results showed that treated pepper plants flowered 20 days earlier and yielded up to 213% more fruit than untreated controls. Altogether the data suggest that exposure of young plants to *C. sphaerospermum* produced VOCs may provide a useful tool to improve crop productivity.

## Introduction

Innumerable bacterial and fungal microorganisms colonize various habitats and can influence the survival of plants. Many such microorganisms promote plant growth through mutualistic interactions which have been the subject of intensive studies for more than 150 years (Whipps, 2001; Berg, 2009). In general, beneficial microorganisms are categorized into a number of groups depending on their habitats and functional roles. They include organic matter decomposers, nitrogen fixing bacteria, mycorrhizal fungi, detoxifiers, mutualistic endophytes, and pathogen-antagonists (Barea et al., 2005). These microbes produce and release unique proteins, enzymes, antibiotics, phenolics, lipids, carbohydrates, and phytohormones improving the availability of essential nutrients and plant growth. In addition, they are capable of activating plant defenses and protecting against biotic and abiotic stresses (Wardle et al., 2004; Barea et al., 2005; Saharan and Nehra, 2011; De-la-Peña and Loyola-Vargas, 2014). Over the past several decades, concerted efforts have focused on the study of plant–microbe symbiotic relationships and endophytic interactions to elucidate plant growth promoting (PGP) activities of beneficial microorganisms (Barea et al., 2005; Berg and Smalla, 2009; Lambers et al., 2009; Saharan and Nehra, 2011; Ahemad and Kibret, 2014; De-la-Peña and Loyola-Vargas, 2014). Consequently, a wide range of microbial inoculants and biocontrol products were developed and marketed as biopesticides, plant strengtheners, phytostimulators, and biofertilizers to improve soil conditions, increase crop productivity and control soil borne pests while reducing reliance on agrochemicals. These products have the potential to encourage environmentally friendly and sustainable cultivation practices and protect biodiversity (Thakore, 2006; Berg, 2009; Baez-Rogelio et al., 2016).

The utilization of current microbial products designed to be applied to the rhizosphere or as inoculants for PGP commonly suffer from unpredictability and inconsistency (Berg, 2009; Ahemad and Kibret, 2014). Changes in soil conditions due to tillage and erosion, extreme weather events and capricious mutualistic interactions amongst microorganisms outside or within host plants can influence the biochemical, physiological and metabolic activities of microbial community and the survival of beneficial microorganisms (De Souza et al., 2015; Baez-Rogelio et al., 2016). A number of studies revealed that some beneficial microorganisms under certain growth and environmental conditions can also produce phytotoxic substances such as hydrogen cyanide (HCN) and other nitrogen and sulfur compounds that can adversely affect plant growth (Kremer and Souissi, 2001; Weise et al., 2013; Nadeem et al., 2014). Clearly, a better understanding of the responses of beneficial microorganisms to their environments will help the development of sustainable strategies that can maximize the effectiveness of microbial products and minimize the deleterious effects on plant growth.

Recently, research activities in the utilization of microorganisms that enhance plant growth via microbial volatile organic compounds (MVOCs) have been intensified based on the premise that they might provide an eco-friendly, cost-effective and sustainable strategy to benefit agriculture (Kanchiswamy et al., 2015a,b; Li et al., 2016). MVOCs are small (<300 Da) and vaporous/gaseous semiochemicals with relatively high vapor pressures, low boiling points and high levels of lipophilicity (Schulz and Dickschat, 2007; Lemfack et al., 2014). These compounds are mostly organic/hydrocarbon in nature (Audrain et al., 2015). Microorganisms from many bacterial and fungal species and genera are able to synthesize a plethora of MVOCs with various biological functions. According to a recent tally, 400 out of 10,000 microorganisms have been described for their ability to produce up to 1,000 MVOCs (Schulz and Dickschat, 2007; Lemfack et al., 2014; Peñuelas et al., 2014; Kanchiswamy et al., 2015b; Li et al., 2016). However, among the complex blends of MVOCs, only a handful have been demonstrated to function in PGP. For instance, 141 MVOCs were detected from 20 strains and 11 species of *Trichoderma* examined. Yet, only 18 compounds or 11% of the detectable pool were able to promote plant growth, whereas the remaining majority compounds were either neutral or inhibitory to plant growth (Lee et al., 2016). In some cases, phytopathogenic microorganisms are also capable of emitting PGP MVOCs but their positive activities are often subdued by strong phytotoxic substances and parasitic or pathogenic behaviors (Sánchez-López et al., 2016b; Cordovez et al., 2017). While specific correlation between microbe-derived volatiles and plant responses requires further examination and the mechanisms of action remain unknown in most cases, a growing number of studies have linked PGP MVOCs to diverse mechanisms. These include regulation of photosynthesis, starch accumulation, phytohormone homeostasis/signaling and cell expansion, activation of innate immunity and abiotic stress responses, reprogramming of developmental controls for promoted reproductive growth, and even altering the behaviors of other microorganisms through inter- and intra- kingdom microbial interactions (Ryu et al., 2003, 2004; Zhang et al., 2007; Minerdi et al., 2011; Pieterse et al., 2014; Bitas et al., 2015; Sánchez-López et al., 2016a,b; Cordovez et al., 2017; Farag et al., 2017; Tahir et al., 2017).

Despite these studies, challenges remain in applying MVOC-mediated PGP technology to crop production. More research is needed to identify and fully exploit novel MVOC-emitting microorganisms and develop effective deployment strategies (Ahmad et al., 2008; Berg, 2009; Kanchiswamy et al., 2015a; Turner and Meadows-Smith, 2016). In this study, we report on the discovery of a non-pathogenic airborne fungus *Cladosporium sphaerospermum*, strain TC09 that strongly stimulates plant growth through exposure *in vitro*. By using tobacco (*Nicotiana tabacum*) and pepper (*Capsicum annuum*) as models, conditions for fungal exposure and fungal cultivation to achieve optimal plant growth stimulation were investigated. We demonstrated growth stimulation, early flowering and fruit yield increases following a relatively short duration of *in vitro* MVOC exposure at seedling stage. Comparative transcriptome analysis was conducted to reveal differential gene expression associated with promoted plant growth and development. PGP activities following *in vitro* exposure using related fungal species and isolates were also compared.

## Materials and Methods

### Culture Media

Premixed medium powder containing basal salts and vitamins of MS medium (Murashige and Skoog, 1962) were purchased from PhytoTechnology Laboratories (Cat No. M519, Overland Park, KS, United States). For *in vitro* culture of plants, full strength MS medium supplemented with 30 g/L sucrose (Sigma-Aldrich, St. Louis, MO, United States, S5391) was prepared with pH adjusted to 5.8 using 1N KOH prior to addition of 7 g/L gelling agar (Sigma, A7921). The medium was then autoclaved at 121°C for 20 min and cooled down to 45°C prior to dispensing to Magenta GA7 vessels (100 ml per vessel) and 15 × 100 mm Petri dishes (30 ml/Plate).

### Species Identification of TC09

TC09 was an environmentally isolated strain of unknown fungus and was subsequently deposited at the Agricultural Research Service Culture Collection (NRRL) with an assigned #: NRRL 67603. To determine the species identity, single spore cultures were transferred to MS medium in Petri plates and incubated at 25°C. Fungal spores were collected from the cultures and kept in a 1.5 ml microcentrifuge tube at -20°C. Genomic DNA was isolated using the DNEasy Plant Mini Kit (Qiagen, Hilden, Germany). Briefly, conidia were removed from the freezer and liquid nitrogen was added to the microcentrifuge tube. The conidia were ground to a fine powder using a motorized pestle mixer (VWR Pellet Mixer, VWR, Intl., Radnor, PA, United States). The DNA was isolated following the manufacturer’s protocol with one exception; in the final step, DNA was eluted from the spin column using 100 μL hot (65°C) nuclease-free water. DNA concentration was determined using a Qubit^®^ 2.0 fluorometer and the dsDNA HS Assay Kit (Life Technologies, Carlsbad, CA, United States). Polymerase chain reaction (PCR) was performed with the genomic DNA as a template. The internal transcribed spacer 1 (ITS1) and ITS2 regions associated with the 5.8S ribosomal RNA (rRNA) gene in fungal organisms were targeted for species identification. Two reactions using primer pairs ITS1/2 and ITS3/4, respectively, were conducted in a Bio-Rad thermocycler using 30 reaction cycles each consisting of 94°C for 1 min, 60°C for 1 min, and 72°C for 3 min. Sequences of the primer pairs are as described by White et al. (1990) (Table 1). Both amplicons were visualized on a 0.8% agarose gel stained with ethidium bromide (EtBr) following electrophoresis. DNA products in single bands were purified from isolated gel blocks using Qiagen PCR Clean Up kit and DNA concentrations were quantified using a Nanodrop spectrophotometer. Purified DNA Products were subject to Sanger sequencing by Eurofins MWG Operon (KY, United States) and sequence data were analyzed using Geneious software (Biomatters Inc., Newark, NJ, United States).

**Table 1:** Oligonucleotide primers and amplicons targeting the internal transcribed spacer 1 (ITS1) and ITS2 regions associated with the 5.8S ribosomal RNA (rRNA) gene in fungi.

Sequence type	Sequence name	Sequence 5′ to 3′	Nucleotide
Forward primer	ITS1	TCCGTAGGTGAACCTGCGG	19
Reverse primer	ITS2	GCTGCGTTCTTCATCGATGC	20
Forward primer	ITS3	GCATCGATGAAGAACGCAGC	20
Reverse primer	ITS4	GGAAGTAAAAGTCGTAACAAGG	24
Amplicon	ITS1/2	GGCCGGGGATGTTCATAACCCTTTGTTGTCCGACTCTGTTGCCTCCGGGGCGACCCTGCCTTTTCACGGGCGGGGGCCCCGGGTGGACACATCAAAACTCTTGCGTAACTTTGCAGTCTGAGTAAATTTAATTAATAA	138
Amplicon	ITS3/4	TTCAGTGAATCATCGAATCTTTGAACGCACATTGCGCCCCCTGGTATTCCGGGGGGCATGCCTGTTCGAGCGTCATTTCACCACTCAAGCCTCGCTTGGTATTGGGCGACGCGGTCCGCCGCGCGCCTCAAATCGACCGGCTGGGTCTTCTGTCCCCTCAGCGTTGTGGAAACTATTCGCTAAAGGGTGCCACGGGAGGCCACGCCGAAAAACAAACCCATTTCTAAGGTTGACCTCGGATCAGGTAGG	249


To determine phylogenetic relationships, DNA sequences of 145 related fungal species/isolates with over 95% homology to the consensus sequence of the ITS3/4 primer pair-amplified PCR products were retrieved via a BLAST search of GenBank database. The sequences were aligned and analyzed using default settings of the Phylogeny Module of CLC Genomics Workbench software program (Version 10, Qiagen, Redwood City, CA, United States) with Neighbor-Joining method with 1,000 bootstrap replicates.

For microscopic examination, TC09 was grown on PDA (Potato dextrose agar) or MS media for 7–10 days at 22°C under continuous light. Squares of transparent adhesive tape (Scotch Magic tape, 3M, St. Paul, MN, United States) were gently placed along the edge of the colony with forceps. Tape squares were removed from the colony margin and stained for 20 min with 1% aqueous Calcofluor white M2R (Fluorescent brightener 28, Sigma, St. Louis, MO, United States). Tape squares were gently rinsed in sterile distilled water and mounted between drops of 50% glycerol under a glass cover slip. The cover slip was affixed in place using clear nail polish. Mounted specimens were visualized through confocal microscopy (Zeiss LSM-800, Carl Zeiss AG, Oberkochen, Germany) and images were captured using the manufacturer software.

### Assay of TC09 Promotion Activities on Tobacco Plant Growth

Tobacco seeds (*Nicotiana tabacum* cv. “Samsun”) were surface-sterilized by rinsing briefly in 95% ethanol and then immersed in 20% (v/v) bleach containing 8.25% w/v sodium hypochlorite with constant agitation for 10 min. After 3× rinse with sterile water, seeds were then spread evenly on the surface of MS medium in Petri plates. The plates were sealed with plastic wrap and maintained at 25°C under 16-h light (50 μmol m^2^ s^1^) and 8-h dark photoperiods for 6 days. Seedlings with fully expanded cotyledons were selected based on a uniform plant size and an appearance free of abnormality (∼80–90% of sowed seeds).

TC09 inoculant in aqueous conidial suspension was prepared by first culturing the fungal conidia on MS plate for 1 week followed by collecting conidia in sterile 0.01% Triton X-100/water solution and adjusting density to 10^5^ conidia per ml prior to use or storage at -80°C. For initial experiments, fungal cultures grown in Eppendorf tubes were placed inside the plant tissue culture vessel but physically separated using sealed biological filters that restrict the movement of conidia but not volatiles. First, aliquots of 300 μl warm MS medium were poured into 1.5 ml Eppendorf microcentrifuge tubes. Tubes were positioned diagonally to form a slant surface while cooling in laminar flow hood. Ten microliters of an aqueous conidial suspension of TC09 was transferred onto the surface of the medium in each tube and thus inoculated tubes were plugged with a sterile aerosol substance- and liquid-resistant filter (Rainin #17001945, Mettler Toledo, Oakland, CA, United States). Two tubes were directly inserted at separate corners of a Magenta GA7 vessel containing three tobacco seedlings each. The vessels were then sealed with plastic wrap and maintained under light conditions with a 16-h photoperiod at 25°C. Plant growth was monitored and compared to controls grown without fungal cultures.

In subsequent experiments open-end culture tube closures (Sigma C5791) were used to reduce premature build-up of condensation water on the surface of the fungal mycelium as found in above microcentrifuge tube setting. Each sterile closure contained 5 ml semi-solid MS medium followed by addition of 10 μl of TC09 suspension as inoculant. One inoculated closure was then placed in each Magenta GA7 vessel that contained three tobacco seedlings for fungal exposure treatment. For controls, a MS-containing closure without fungal inoculant was added. Vessels were placed under above-mentioned light conditions at 25°C. To measure plant growth promotion under *in vitro* conditions, a 20-day exposure duration was employed which was comparable to previous studies using tobacco (Paul and Park, 2013; Lee et al., 2016).

Three plants were grown in each replicate vessel and three vessels were used per treatment. The experiment was conducted three times unless otherwise stated. Plant growth was determined by measurement of major growth parameters including total plant height, total plant fresh weight, stem length, number of traceable leaves, root system length, and length of largest leaf. Data were analyzed by using SAS statistics program with standard statistical approaches including standard error, *t*-test (two means), ANOVA and Tukey’s HSD mean separation test (multiple means) with a statistical significance level *P* < 0.05.

### Effect of TC09 Exposure Duration and Sugar Concentration

For experiments to test the effect of exposure time under *in vitro* conditions, control tube closures with medium-only (CK) and those with fungal cultures were placed in vessels containing tobacco seedlings. Fungal cultures were then removed after an incrementally extended exposure duration from 1, 4, 10, or 20 days. Vessels containing medium only were treated as 0 day and served as a control. All plants were allowed to grow continuously in the vessels until 20 days after introduction of fungal cultures when growth was measured. The experiment was conducted two times.

A total of six sucrose concentrations including 0, 5, 10, 20, 30, and 40 g/L were incorporated in MS medium and used to culture tobacco seedlings. Introduction of TC09 cultures in tube closures to Magenta GA7 vessels was carried out as aforementioned. After a 20-day fungal exposure, plants were collected, measured and analyzed as described above. Experiments were conducted two times.

### Plant Growth Under *ex vitro*/Soil Conditions in the Greenhouse

*In vitro* grown plants following fungal exposure were transplanted to potting soil mix (Metro Mix 360, Sun Gro Horticulture, Canada) and maintained in a greenhouse to monitor growth. Soil mix was pre-sterilized by autoclaving at 121°C for 90 min prior to use. Plants were maintained in a greenhouse. Six to ten individual tobacco plants from each treatment and untreated controls were transplanted for each test and experiments were conducted three times. Plant growth and development were evaluated via periodic measurement of plant height and total leaf numbers over the course of 70 days beginning from the time the seeds were sown. Experimental data were analyzed using standard statistical approaches as mentioned above.

### Effect of TC09 Growth Media on Plant Growth Promotion

A total of six media were examined for fungal cultivation including MS (Murashige and Skoog, 1962), PDA (potato dextrose agar), Czapek (CYA, Czapek-DOX Yeast agar), Malt (Malt extract agar), yeast (Yeast extract extract) and Hutner’s medium. Formula and preparation procedures for the last five culture media were described previously (Sinclair and Dhingra, 1995). Organic potatoes were purchased locally and used to prepare potato infusion and PDA medium. For these experiments, tobacco and closure-mediated method for fungal exposure were employed. Final determination of plant growth was made at the end of an exposure duration of 20 days. There were three plants per replicate vessel and three vessels per treatment. The experiment was repeated twice.

### Effect of TC09-Related Fungal Species/Isolates on Plant Growth Promotion

Comparative assays were performed to determine the effectiveness of exposure *in vitro* using various species and isolates belonging to *Cladosporium* that were kindly provided by Dr. Frank M. Dugan of USDA (59 Johnson Hall, WSU, Pullman, WA, United States). These include *C. sphaerospermum* NRRL8131, *C. cladosporioides* 113d, *C. asperulatum* 208db, *C. subtilissimum* WF99-209, *C. cladosporioides* W99-175a, and *C. macrocarpum* Clad ex Phyl 8. Tobacco plants were used for growth comparison using MS medium for fungal culture. Three replicate vessels with three plants each were used for each treatment. The experiment was repeated twice.

### Comparative Transcriptome Analysis of Tobacco Plants Following Exposure to TC09

Transcriptome analysis was carried out using total RNA isolated from tobacco seedlings grown under *in vitro* conditions with or without TC09 exposure for 10 days. For RNA isolation, shoot tips carrying the apical meristem and three to four terminal leaves were collected from each replicate plant, placed in 1.5 ml microcentrifuge tubes, and immediately immersed in liquid nitrogen. Samples were pulverized into a fine powder using Geno/Grinder 2010 (SPEX Sample Prep, Metuchen, NJ, United States). Total RNA was then extracted from 100 mg tissue powder using the RNeasy^®^ Plant Mini Kit per manufacturer’s instructions (Qiagen, Redwood City, CA, United States). There were four biological replicates from each treatment. RNA sequencing was performed by Genewiz via Illumina HiSeq platform (50 bp single-end reads) (South Plainfield, NJ, United States). Raw sequences were paired, trimmed and filtered to obtain quality reads. Reads were mapped to the *Solanum lycopersicum* reference genome with CLC Genomics Workbench (version 10, Qiagen, Redwood City, CA, United States) using default parameters (The Tomato Genome Consortium, 2012). Differentially expressed genes (DEGs) were identified using false discovery rate (FDR) ≤ 0.05. Genes were annotated per referenced tomato (*S. lycopersicum*) transcriptome dataset available on https://solgenomics.net and associated gene ontology (GO) terms. The RNAseq data was deposited to NCBI GEO under reference ID GSE120288.

### Effect of TC09 Exposure *in vitro* on Pepper Plant Growth and Production

Two different varieties of pepper (*Capsicum annuum*), cayenne pepper ‘Long Thin’ and sweet bell pepper ‘Minisweet Pepper Mixed,’ were tested to determine growth response to TC09 exposure *in vitro*. Pepper seeds were acquired from commercial source (Plantation Product LLC., Norton, MA, United States) and sterilized by previously described method prior to *in vitro* cultivation or seeded directly in the soil as controls. Germinated pepper seedlings were exposed to closure-contained TC09 while culturing on MS medium in GA7 vessels for 20 days and then transplanted to soil in 8-inch pots. Plants were maintained in the greenhouse using standard management practice. Plant growth and fruit production were monitored continuously until fruit ripening. In these experiments each treatment consisted of four to five individual plants. Experiments were conducted two times each in a similar seasonal span from late winter to early spring in 2017 and 2018, respectively.

## Results

### Isolation and Species Identification of TC09 Fungus

An airborne fungal contaminant on a tissue culture plate containing tobacco seedlings growing on MS medium was isolated and single-spore purified based on the preliminary visual observation that plants in the contaminated plate grew much larger than comparable plants on uncontaminated plates. To identify the species, DNA was extracted and the internal transcribed spacer was amplified by PCR and subsequently sequenced. The sequence of the two fragments generated from the primer pairs are specified in Table 1.

Upon comparison with existing sequences, the 138 bp ITS1/2 amplicon was 100% identical to a GenBank accession KU926349.1 belonging to *Cladosporium sphaerospermum* isolate UACH-124. The 249 bp ITS3/4 amplicon was 100% identical to a GenBank accession KX982238.1 of *Cladosporium sphaerospermum* strain 7. Phylogenetic analysis of 148 sequences homologous to the ITS3/4 amplicon that were available in GenBank showed that TC09 clusters with known isolates of *C. sphaerospermum* (Supplementary Figure S1).

To confirm the results of genetic identification, TC09 was cultured on PDA medium, which is commonly used as a standard condition for fungal species identification. TC09 colonies grew to 20 mm and 35 mm in diameter during 7 and 14 days, respectively, on PDA at 20°C.=. Colonies were olivaceous-gray in the center and olivaceous to iron-gray in the outer region (Supplementary Figure S3A). On the reverse they were iron-gray and turned almost blackish at 14 days. Conidiophores were not much differentiated, occasionally branched with conidia produced in branching chains with variable shapes with smaller size toward the apex (Supplementary Figures S3B,C). Intercalary conidia were 1.7–3.2 × 2.4–8.0 μm and terminal conidia 1.9–2.5 × 2.1–2.9 μm. Hyphae was 2.6–3.2 μm wide, sparsely to profusely branched at 45–90° angles, distinctly septate with cell length averaging 17.5 μm and ranging from 12.4 to 27.3 μm (Supplementary Figure S3D). Collectively, the morphological results were consistent with TC09 being an isolate of *C. sphaerospermum* as described by Dugan et al. (2008) and Ababutain (2013).

### Stimulation of Plant Growth Is due to *in vitro* Exposure to TC09

**FIGURE 1:**
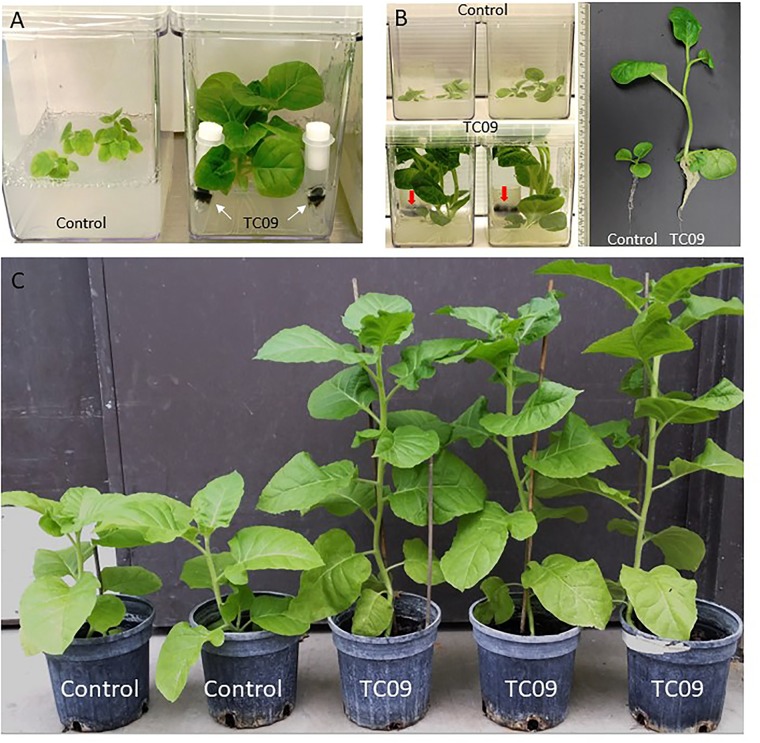
Growth promotion of tobacco by exposure to TC09. **(A)** Control tobacco plants without fungal exposure (left) and plants exposed to physically separated TC09 contained in filter-sealed microcentrifuge tubes (right). Arrows indicated the fungus with dark-colored mycelium. Photograph was taken 10 days after fungal exposure. **(B)** Tobacco plants exposed to TC09 contained in open-end tube closures. Images were taken 20 days after introduction of fungal cultures. Left and upper row: control plants without TC09 exposure in triplicates per vessel, Left and lower row: plants with exposure to fungal cultures in triplicates per vessel. Arrows indicate TC09 mycelium in closure. Right panel: representative plants of control (left) and TC09-exposed treatment (right). **(C)** Evaluation of plant growth in greenhouse following *in vitro* seedling treatment with or without TC09 exposure. Plants with (right three) or without (left two) exposure to TC09 for 20 days were transplanted to soil and maintained in greenhouse using standard management practices. Photograph was taken 70 days after seed sowing.

Since the fungal mycelium and tobacco plants appeared to be physically separated in the original contaminated culture plate, possible involvement of MVOCs in plant growth promotion was investigated. Tobacco plants were tested with and without the inclusion of biological filter-sealed Eppendorf tubes containing TC09 in Magenta culture vessels. In this manner, the plants were exposed only to volatiles produced by the fungus. Visual observation indicated that plants with fungal exposure for 10 days exhibited more vigorous growth and thicker stems, larger leaves, and a more robust root system relative to plants without fungal exposure (Figure 1A). No conidia from the filter-sealed Eppendorf tubes were visually detected in either the culture vessel headspace or plant culture medium.

Next, plant growth stimulation was quantified using a 20-day exposure duration and open-end culture tube closures to prevent excessive condensation in the fungal culture. Exposed plants grew larger and filled up the headspace of culture vessels, produced elongated stems with larger diameters and longer internodes as well as a larger root systems as compared to controls (Figure 1B). Plants were subsequently transplanted to soil and growth differences were maintained (Figure 1C).

**FIGURE 2:**
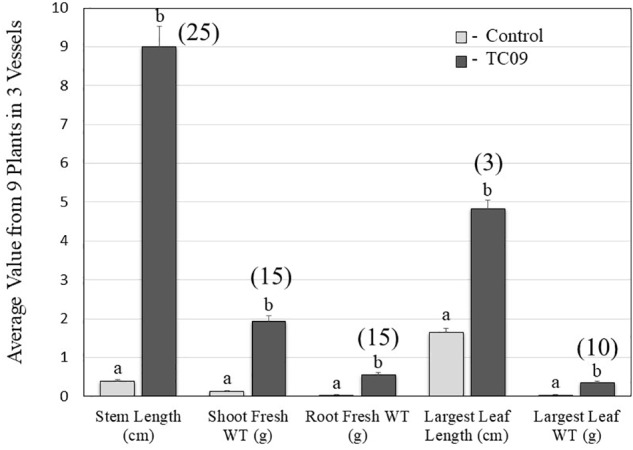
Quantification of five growth parameters of control and TC09-exposed *in vitro* tobacco plants. Six-day-old tobacco seedlings following seed sowing were exposed to medium-containing closures (control) or fungal culture containing closures for 20 days and then subject to measurement to determine growth status. Values represent means obtained from triplicate vessels for each treatment. Each vessel contains three individual plants. Data in shaded bars from TC09-exposed plants were converted into fold increase over corresponding values from control plants and labeled in parentheses. Standard errors of the means were marked as vertical lines. Paired treatments sharing a letter on top of bars are not significantly different at *P* > 0.05 based on Tukey’s test.

**FIGURE 3:**
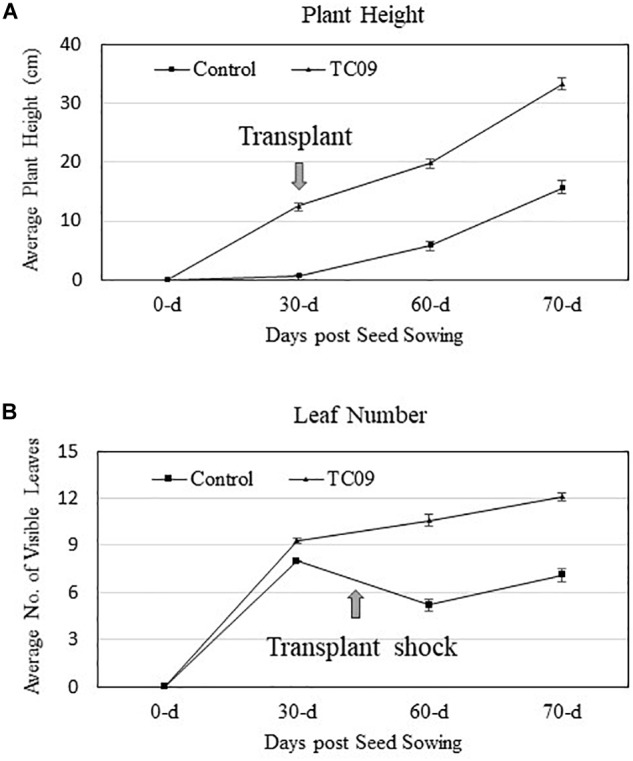
Stem growth and leaf production of greenhouse-grown tobacco plants with or without *in vitro* exposure to TC09. Thirty-day-old plants with or without prior fungal exposure for 20 days were transplanted to soil and maintained in greenhouse using standard management practices. Plant height and the number of visible fully expanded leaves per plant were determined periodically. **(A,B)** Graphs denote changes in height and leaves, respectively, during the monitored growth period. Values represent average data from nine replicated plants per treatment and two repeated experiments.

As compared to controls, the individual plants following 20-day *in vitro* exposure treatment exhibited the following growth increase: 25-fold for stem length, 15-fold for shoot biomass, 15-fold for root biomass, 3-fold for base-to-top length of largest leaf, and 10-fold for largest leaf biomass (Figure 2). Results from repeat experiments essentially gave rise to similar increased levels of plant growth with exposure to the fungal culture (data not shown). When planted in soil, the treated plants maintained a near twofold increase in height and in leaf number at 70 days post seed sowing relative to controls (Figures 3A,B).

### Effect of TC09 Exposure Duration on Plant Growth

**FIGURE 4:**
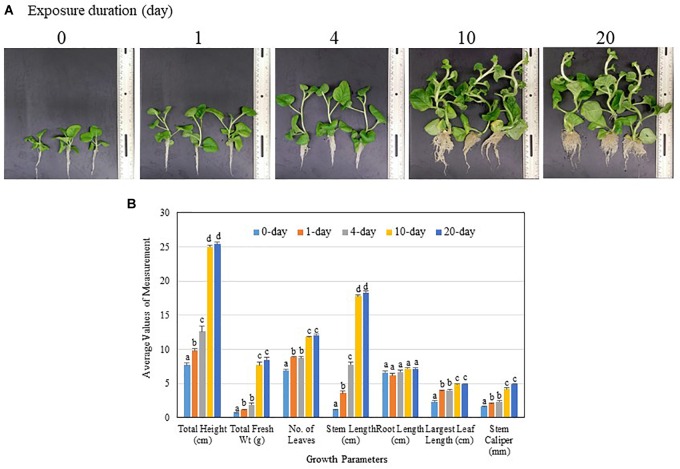
Effect of MVOC exposure duration on tobacco growth under *in vitro* conditions. Seedlings 6 days after seed sowing were exposed to TC09 for specified durations from 1, 4, 10 to 20 days as TC09 exposure treatments or maintained without MVOC exposure (0 day as control). Plants were then allowed to grow continuously until the time for the last treatment was met, i.e., 20 days of exposure. **(A)** Representative plants from each treatment taken at the end of 26-day cultivation. Exposure durations were labeled on each image. **(B)** Values of seven growth parameters of control and TC09-exposed *in vitro* tobacco plants determined at the end of 26-day cultivation using various exposure duration. Bar values represent means from triplicate culture vessels for each treatment with each vessel containing three plants. Lines on top denote standard errors. Means sharing a letter in the group label are not significantly different at *P* > 0.05 according to Tukey’s test.

The potency of PGP activity of TC09 was ascertained using incrementally extended exposure durations. Visual inspection at the end of a 26-day culture period post seed sowing indicated that 1-day exposure of tobacco seedling to the freshly set-up TC09 culture was sufficient to double the plant size as compared to plants without exposure (0 day vs. 1 day) (Figure 4A). Plant growth was incrementally increased after 4-, 10-, and 20-day exposures with 10 days being maximal (Figure 4A). Based on measurements of major growth parameters, 10-day or 20-day exposure duration led to increase in stem length, stem caliper (diameter), and total plant biomass 16-, 3-, and 12-fold, respectively, over the control without TC09 exposure (Figure 4B). Repeated experiments yielded similar results (data not shown).

### Effect of Sucrose Concentration and TC09 Exposure on Plant Growth

**FIGURE 5:**
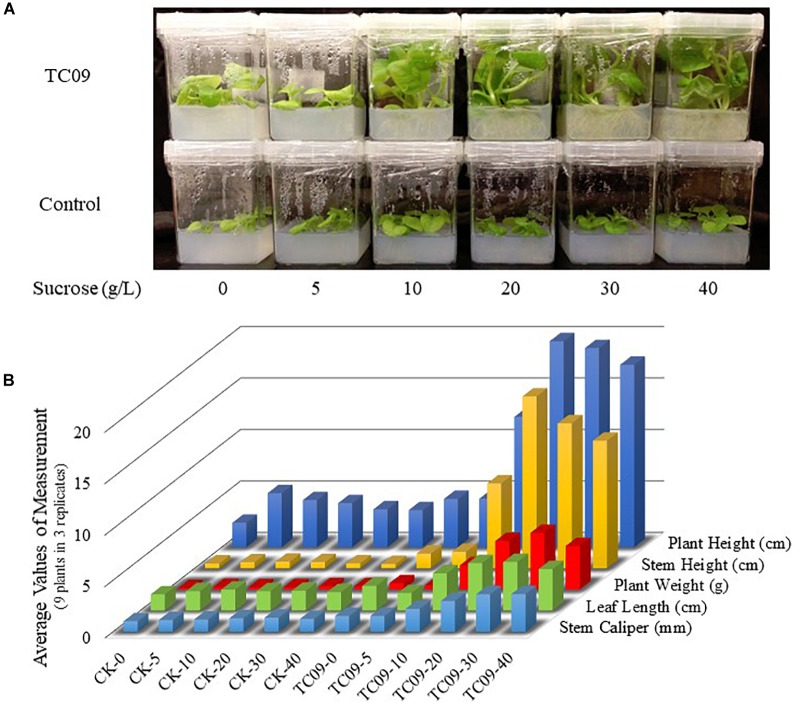
Effect of sucrose concentration and TC09 exposure on tobacco growth under *in vitro* conditions. Seedlings 6 days post seed sowing were cultured on MS medium containing various concentrations of sucrose and exposed to freshly prepared TC09 culture in tube closure. **(A)** Plants were imaged 20 days after inset of fungal exposure. **(B)** Bars represent average values of growth parameters from three plants in each culture vessel with three replicate vessels per treatment.

To investigate whether carbon source availability was a contributing factor for PGP activity, various concentrations of sucrose were tested in conjunction with TC09 exposure for 20 days. Without fungal exposure, tobacco plants did not show any measurable increase in growth rate in response to increasing concentrations of sucrose in the medium (Figure 5). When plants were exposed to TC09, only minimal plant growth promotion was observed in media containing 0 and 5 g/L sucrose whereas significant growth promotion was achieved when plants were maintained on a medium containing > 10 g/L sucrose (Figure 5). These results were also confirmed in a repeat experiment (data not shown).

### Effect of Fungal Culture Media on PGP Activities of TC09

**FIGURE 6:**
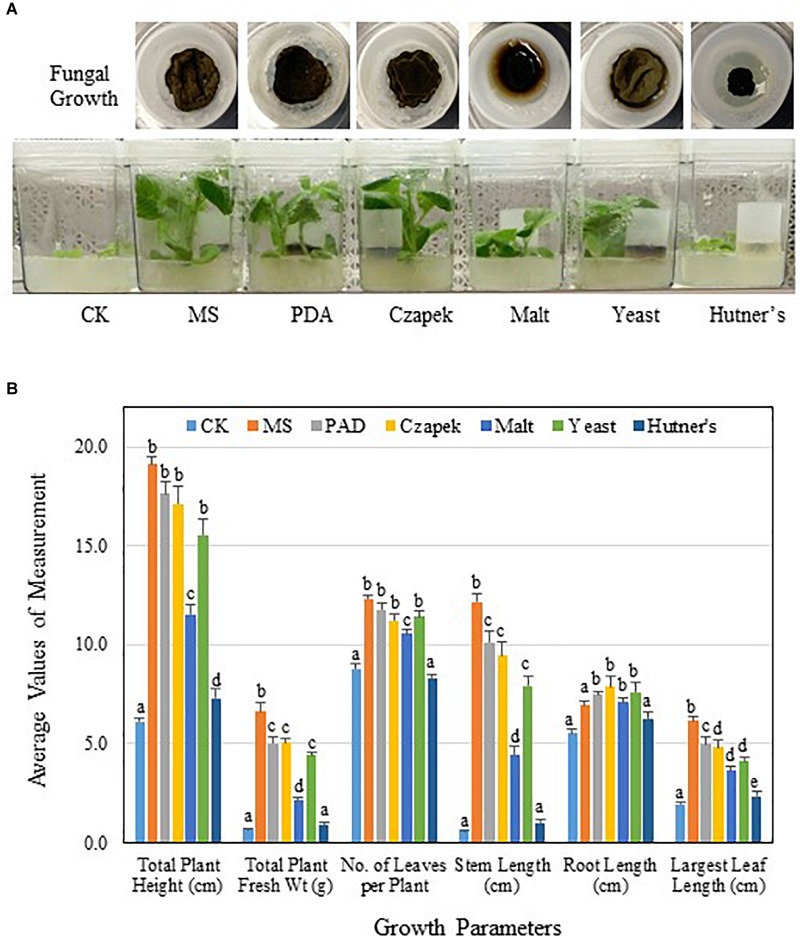
PGP activity of TC09 affected by fungal culture media. **(A)** Fungal growth or hyphal development in various culture media and corresponding plant growth promotion. TC09 cultures were initiated in closures and used for exposure to 6-day-old tobacco seedlings according to described procedure. Images were taken 2 weeks after exposure initiation. Abbreviated names of fungal culture media are labeled. **(B)** Major growth parameters of control and TC09-exposed *in vitro* tobacco plants using various fungal culture media. Measurements were made 20 days after fungal exposure. Values represent means of three plants each of triplicate vessels per treatment. Standard errors of the means were labeled as vertical lines. Means sharing a letter in the group label are not significantly different (*P* > 0.05) according to Tukey’s test.

Initial PGP activity of TC09 was characterized using MS medium. Thus, to investigate the influence of culture conditions, several common fungal media were also tested. Variations in visual size and morphology of TC09 mycelium were discernable among various culture media used (Figure 6). Noticeable differences in the level of PGP activity of fungal cultures on various media were also observed (Figure 6). Twenty days post treatment, plants were measured to determine growth differences including total plant height, total plant biomass, stem length, and length of largest leaf. Results revealed that the order of growth stimulation level from highest to lowest among tested fungal culture media was as follows: MS>PDA>Czapek>Yeast>Malt>Hutner’s (Figure 6B). Hutner’s medium contained rich vitamins and the same amount of sucrose as MS medium, but it showed the lowest levels of TC09 growth and PGP activity. On the other hand, Malt and Yeast media, both with reduced PGP activities as compared to MS, encouraged vigorous TC09 growth with comparable colony sizes to that of other media except for Hutner’s medium. In addition, Malt medium seemed to increase hydrophilicity of the mycelium of TC09 and promote hyphal penetration into culture medium (Figure 6A). We conducted a repeat experiment and observed an identical trend of growth stimulation associated with various fungal media (data not shown).

### Comparison of PGP by TC09 and Other Related Fungi

**FIGURE 7:**
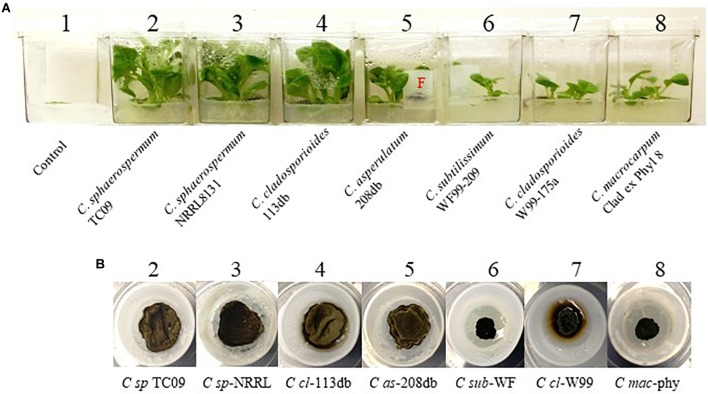
Comparison of growth stimulation activity of various isolates/species of *Cladosporium*. **(A)** Tobacco plants exposed for 15 days to seven isolates/species of *Cladosporium* along with controls without exposure (#1). Names of species and isolates of *Cladosporium* are labeled. F denotes fungal culture in a closure. **(B)** Mycelium of various compared fungi in closures containing MS medium.

To investigate whether the observed PGP activity was unique to TC09-or common among related fungal species, we compared a total of seven *Cladosporium* species/isolates for their ability to promote *in vitro* tobacco growth. All strains exhibited PGP activity as compared to control without fungal exposure, although there were measurable differences among the species/isolates (Figure 7A). After 20 days of exposure, TC09 gave the highest level or PGP activity in five out of seven growth parameters measured (Table 2). Exposure to *C. sphaerospermum* NRRL8131 produced the longest stems among all compared fungi (Table 2). Colony morphology and growth pattern of various *Cladosporium* fungi when cultured on MS medium were also noticeably varied (Figure 7B). Four of the tested fungi including *C. sphaerospermum* TC09 (1), *C. sphaerospermum* NRRL8131 (2), *C. cladosporioides* 113 db (3) and *C. asperulatum* 208 db (4) produced mycelium in a similarly large diameter and dark greenish brown color. The remaining fungi including *C. subtilissimum* WF99-209 (5), *C. cladosporioides* W99-175a (6) and *C. macrocarpum* Clad ex Phyl 8 (7) formed smaller, black colonies. *C. cladosporioides* W99-175a (6) is the only fungus which produced dark brownish compounds that penetrated into the culture medium, whereas all other fungi demonstrated hydrophobicity of the mycelium on MS medium (Figure 7B). Subsequently, we conducted a repeat experiment that yielded similar results (data not shown).

**Table 2:** Fold increase over control of growth parameters from tobacco plants exposed to MVOCs emitted by various *Cladosporium fungi*.

Growth parameters	Item	Species/isolates of *Cladosporium*							
		
		CK	*C sp* TC09	*C sp* NRRL	*C cl* 113 db	*C as* 208 db	*C sub* WF	*C cl* W99	*C mac* phy
Total plant	Average (SD)	4.63 (0.880)	16.92 (0.657)	17.44 (1.079)	17.07 (2.389)	16.66 (1.316)	10.71 (1.263)	12.19 (0.686)	8.78 (1.241)
height (cm)	Fold over CK^5^	0	2.7	**2.8**	2.7	2.6	1.3	1.6	0.9
	SD label^1^	a	b	b	b	b	c	c	d
Total plant	Average (SD)	0.05 (0.015)	6.22 (0.829)	4.21 (1.063)	4.76 (0.595)	3.65 (1.170)	1.18 (0.311)	1.50 (0.709)	1.18 (0.179)
fresh Wt (g)	Fold over CK	0	**123.4**	83.1	94.1	72.0	22.7	29.1	22.6
	SD label	a	b	c	c	c	d	d	d
No. of leaves	Average (SD)	5.7 (0.707)	10.4 (0.527)	8.9 (0.500)	8.6 (0.500)	9.7 (0.333)	8.7 (0.527)	8.4 (0.527)	7.3 (0.500)
per plant	Fold over CK	0	**0.8**	0.6	0.5	0.7	0.5	0.5	0.3
	SD label	a	b	c	c	b	c	c	d
Stem	Average (SD)	0.30 (0.050)	10.59 (0.764)	11.72 (1.045)	11.20 (1.997)	11.27 (1.054)	4.22 (1.032)	6.04 (0.704)	2.81 (0.562)
length (cm)^2^	Fold over CK	0	34.3	**38.1**	36.3	36.6	13.1	19.1	8.4
	SD label	a	b	b	b	b	c	c	c
Root	Average (SD)	4.33 (0.910)	6.33 (1.067)	5.72 (0.885)	5.89 (0.985)	5.39 (1.179)	6.49 (0.800)	6.14 (0.987)	5.97 (0.837)
length (cm)^3^	Fold over CK	0	**0.5**	0.3	0.4	0.2	0.5	0.4	0.4
	SD label	a	b	a	b	a	b	b	b
Largest leaf	Average (SD)	0.50 (0.087)	5.48 (0.576)	5.02 (0.733)	5.13 (0.572)	4.02 (1.017)	2.70 (0.695)	3.11 (0.622)	3.13 (0.180)
length (cm)^4^	Fold over CK	0	**10.0**	9.0	9.3	7.0	4.4	5.2	5.3
	SD label	a	b	b	b	c	d	cd	cd
Stem	Average (SD)	0.92 (0.136)	4.35 (0.235)	3.44 (0.575)	3.39 (0.317)	3.17 (0.521)	1.74 (0.345)	2.09 (0.416)	2.22 (0.417)
diameter (mm)	Fold over CK	0	**3.7**	2.7	2.7	2.4	0.9	1.3	1.4
	SD label	a	b	c	c	c	d	d	d


*Names of species and isolates are abbreviated and described in the section “Materials and Methods.” Average values were derived from triplicate vessels with three plants each for each treatment. Highest values of fold increase over control (CK) in each category were in bold letters. ^*1*^ Significant difference label generated by Tukey’s mean separation test. Average values sharing a letter within a growth parameter are not significantly different at P = 0.05. Data were averaged from three plants in each replicate vessel and three vessels per treatment. ^*2*^ The length of stem segment measuring from the base of the plant to the top of shoot apex. ^*3*^ The length of root system measuring from the base of the plant to the tip of longest root. ^*4*^ The vertical length of the blade of a largest leaf of each plant excluding petiole. ^*5*^ Fold over CK reflects average value fold increase of treatment over control as follows:*$\text{Fold over CK=}\frac{\text{Average value of treatment-Average value of control}}{\text{Average value of control}}$

### RNAseq Analysis of Differential Gene Expression (DEG) Associated With TC09 Induced Growth Promotion

To assess host responses at the RNA level, tobacco seedlings treated with and without TC09 exposure for 10 days were subject to RNAseq analysis to identify DEGs. The sampling point was chosen based on the consistent levels of plant growth stimulation achieved following 10 days of MOVC exposure (Figure 4). Experimental design included four replicate TC09 treated plants and four untreated control plants sampled after 10 days of exposure. Approximately 20 million quality reads per sample were obtained. A total of 3,562 DEGs were identified consisting of 1,594 up-regulated and 1,968 down-regulated genes (Supplementary Table S1 and Supplementary Figure S2). Overall, the most highly repressed DEGs included reactive oxygen response genes such as those encoding catalases and superoxide dismutases involved in redox balance as well as photosystem functions including photosystem II D1 core genes along with constituents of the cytochrome and ATP synthase complexes. Among the over-represented DEGs were a number of genes involved in cell wall biosynthesis, cell expansion and multiplication. Numerous genes associated with the biosynthesis and response to phytohormones were differentially expressed with those related to auxin, gibberellin, and ethylene being the most abundant.

JA-inducible genes were notably up-regulated as the result of TC09 exposure. These included genes related to herbivore defense including proteinase inhibitors, Kunitz trypsin inhibitor, and *O*-methyltransferases – which catalyze multiple reactions in the biosynthesis of furanocoumarins (FCs) that act as insect repellants (Table 3).

**Table 3:** DEGs, associated major biological functions and expression changes from tobacco seedlings with exposure to TC09 for 10 days.

Annotation	Proposed function	Feature ID	Fold Incr
Auxin response factor 8B	Phytohormone synthesis and regulation	Solyc02g037530.3	2.3
Auxin-regulated IAA17	Phytohormone synthesis and regulation	Solyc06g008590.3	4.3
Auxin-regulated IAA19	Phytohormone synthesis and regulation	Solyc03g120380.3	2.6
Auxin-regulated IAA7	Phytohormone synthesis and regulation	Solyc06g053830.3	2.1
Auxin-regulated IAA16	Phytohormone synthesis and regulation	Solyc01g097290.3	2.1
GRAS family transcription factor	Phytohormone synthesis and regulation	Solyc08g014030.1	4.0
Cytokinin oxidase/dehydrogenase	Phytohormone synthesis and regulation	Solyc04g080820.2.1	4.3
Protease inhibitor/seed storage	Insect Resistance	Solyc01g066910.3	3.6
Kunitz trypsin inhibitor	Insect Resistance	Solyc06g072230.1	2.3
Major latex-like protein	Insect Resistance	Solyc05g046140.3	3.3
Major latex-like protein	Insect Resistance	Solyc05g046160.1	4.0
Cytochrome P450 family protein	Insect R, Coumarins biosynth	Solyc04g080100.3	3.4
Cytochrome P450	Insect R, Coumarins biosynth	Solyc04g083160.2	2.2
*O*-Methyltransferase 3	Insect R, Furanocoumarin biosynth	Solyc10g008120.2.1	1.9
*O*-Methyltransferase	Insect R, Furanocoumarin biosynth	Solyc01g111900.3	3.2
*O*-Methyltransferase-like protein	Insect R, Furanocoumarin biosynth	Solyc12g009110.1.1	3.0
*O*-Methyltransferase	Insect R, Furanocoumarin biosynth	Solyc06g064510.2.1	3.6
*O*-Methyltransferase	Insect R, Furanocoumarin biosynth	Solyc01g068550.2.1	4.0
*O*-Methyltransferase	Insect R, Furanocoumarin biosynth	Solyc10g047520.1.1	9.1
*O*-Methyltransferase	Insect R, Furanocoumarin biosynth	Solyc06g064500.2.1	14.6
Jasmonic acid 1 (JA-1)	ISR activation, JA signaling pathway	Solyc05g007180.3	2.3
Pathogenesis-related thaumatin	Defense	Solyc04g081560.3	4.4
Defensin	Defense	Solyc04g008470.3	4.0
Pathogenesis-related thaumatin	Defense	Solyc02g083790.3	2.3
Pathogenesis-related thaumatin	Defense	Solyc10g084840.2	2.3
Pathogenesis-related thaumatin	Defense	Solyc03g118780.3	2.3
Peroxidase	Defense	Solyc02g094180.3	2.3
Endoglucanase	Defense	Solyc12g055970.2	2.2
UDP-glucose glucosyltransferase	Glycosylation, hormone modulation	Solyc08g062220.2.1	-9.9
UDP-glucose glucosyltransferase	Glycosylation, hormone modulation	Solyc04g016220.2.1	-2.9
UDP-glucose glucosyltransferase	Glycosylation, hormone modulation	Solyc08g006330.2.1	-2.47
UDP-glucose glucosyltransferase	Glycosylation, hormone modulation	Solyc08g006350.2.1	-2.3
UDP-glucosyltransferase family 1 protein	Glycosylation, hormone modulation	Solyc12g042600.1.1	-2.2
UDP-glucose glucosyltransferase	Glycosylation, hormone modulation	Solyc08g006410.2.1	-2.2
Sucrose transporter-like protein	Alternative carbon assimilation	Solyc05g007190.2.1	1.5
Glucose-1-phosphate adenylyltransferase	Alternative carbon assimilation	Solyc01g109790.2.1	1.4
Glucose-6-phosphate translocator	Alternative carbon assimilation	Solyc07g064270.2.1	1.4
Glucose transporter 8	Alternative carbon assimilation	Solyc01g080680.2.1	1.8
UDP-glucose 6-dehydrogenase	Alternative carbon assimilation	Solyc02g088690.2.1	1.5
UDP-D-glucose dehydrogenase	Alternative carbon assimilation	Solyc02g067080.2.1	1.7
UDP-glucose dehydrogenase	Alternative carbon assimilation	Solyc03g115380.1.1	1.8
Cyclin A1	Cell cycle	Solyc11g005090.1.1	1.7
Cyclin-dependent kinase	Cell cycle	Solyc09g065200.2.1	1.8
Cyclin B	Cell cycle	Solyc12g094600.1.1	1.9
Cyclin D	Cell cycle	Solyc12g088650.1.1	2.1
Cyclin D3-1	Cell cycle	Solyc04g078470.2.1	1.8
Cyclin-like protein	Cell cycle	Solyc01g087450.2.1	2.3
Cyclin-dependent kinase regulator Pho80	Cell cycle	Solyc01g089850.2.1	2.6
Cyclin-dependent kinase	Cell cycle	Solyc01g090800.2.1	8.0
Extensin	Cell expansion	Solyc04g071070.2	4.4
Expansin-1	Cell expansion	Solyc06g076220.2.1	1.6
Expansin – pollen	Cell expansion	Solyc12g089380.1.1	1.6
Expansin	Cell expansion	Solyc02g081210.2.1	1.7
Expansin – pollen	Cell expansion	Solyc06g051800.2.1	2.1
Expansin – pollen	Cell expansion	Solyc09g010860.2.1	2.4
Expansin-1	Cell expansion	Solyc10g086520.1.1	2.5
Expansin – pollen	Cell expansion	Solyc00g017230.1.1	2.6
Expansin – pollen	Cell expansion	Solyc10g084780.1.1	2.9
Expansin B1 – pollen	Cell expansion	Solyc10g008440.2.1	3.0
Expansin	Cell expansion	Solyc10g084780.2	3.5
Expansin – pollen	Cell expansion	Solyc02g088100.2.1	4.7


*Fold change in expression levels was calculated based on means of four biological replicates for both control and MVOC-treated plants. Genes were annotated using GO terms and tomato (*Solanum lycopersicum*) reference genes available on https://solgenomics.net.*

Among cyclin-associated genes, cyclin A and B and two cyclin D genes increased twofold to threefold. Two cyclin-dependent kinases (CDKs) genes increased twofold to eightfold, respectively, in expression. In addition, one CDK regulator gene showed a 2.6-fold up-regulated expression. A number of genes encoding auxin regulators and sugar transporters were also up-regulated. Expression of several gylcosyltransferases functioning in glycosylation was reduced 2- to 10-fold (Table 3).

### TC09 Exposure Increases Productivity in Peppers

**FIGURE 8:**
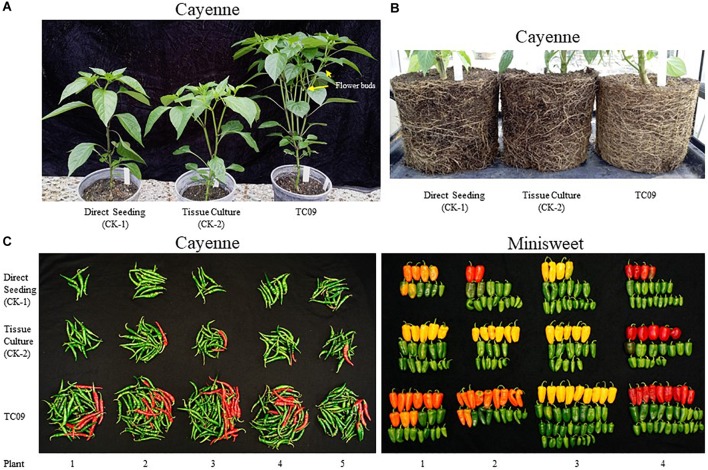
Plant growth and fruit production of pepper (*Capsicum annuum*) varieties cayenne ‘Long Thin’ and sweet bell ‘Minisweet Pepper Mixed’ promoted by exposure *in vitro* to TC09. TC09 exposure for 20 days at seedling stage was identical to that used for tobacco. **(A)** Representative cayenne plants from direct seeding, tissue culture and TC09 treatments at 50 days post seed sowing. **(B)** Representative belowground image of cayenne pepper plants from various treatments at 116 days post seed sowing to depict the density and development of fibrous root mass. **(C)** Lineup of fruits collected from cayenne pepper (left panel, at 116 days post seed sowing) and minisweet pepper (right panel, at 160 days post seed sowing) from all treatments. Fruits in reddish color indicate vine-ripening.

Two varieties of pepper (*Capsicum annuum*) were used to investigate whether exposure to TC09 at the seedling stage could positively influence fruit production in a Solanaceous crop plant. Two independent experiments were carried out for each variety. The pepper seedlings were treated with and without TC09 for 20 days under *in vitro* conditions as described for tobacco. TC09–exposed cayenne and minisweet pepper plants responded to fungal stimulation in a growth pattern similar to tobacco and grew significantly larger than untreated controls that were either started in tissue culture or planted directly to soil. For cayenne pepper, following transplanting to soil, the first flowers among TC09-exposed plants were observed 20 days earlier than control plants along with a denser canopy and more branches (Figure 8A). By 116 days post seed sowing, visual inspection of the root system showed that TC09-exposed plants had more vigorous root growth than both direct soil seeding and tissue culture control plants without fungal exposure (Figure 8B).

**FIGURE 9:**
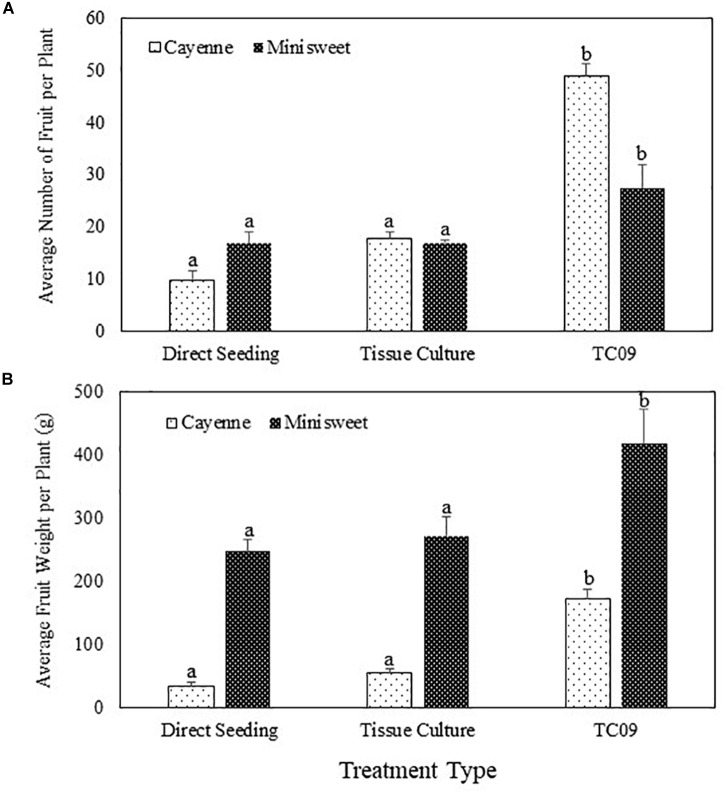
Fruit yield of pepper (*Capsicum annuum*) varieties cayenne ‘Long Thin’ and sweet bell ‘Minisweet Pepper Mixed’ promoted by exposure *in vitro* to TC09. Fruits were collected at 116 days and 160 days post seed sowing for cayenne and minisweet peppers, respectively. Greenhouse tests with these two pepper varieties were carried out separately at different time periods of the year due to space limitations. **(A)** Average number of fruit per plant. **(B)** Average fresh weight of fruit per plant. Standard errors of the means were labeled as vertical lines on top of data bars. Means sharing a letter in the group label are not significantly different (*P* > 0.05) according to Tukey’s test.

Cayenne peppers longer than 1 cm in length were collected from all plants at 116 days post seed sowing. Results showed that TC09-exposed plants had the heaviest fruit load and the most vine-ripened fruits (reddish color) among all treatments (Figure 8C, left panel). Similar patterns of fruit production and development were also observed in minisweet pepper when all fruits were collected at 160 days post seed sowing (Figure 8C, right panel). For cayenne pepper, TC09-treated plants produced on average 49 fruit per plant whereas direct seeding and tissue culture-treated plants only put out on average 10 and 18 fruit per plant, respectively. Likewise, for minisweet peppers, the number of fruits per plant from these three treatments were 28, 17, and 17, respectively (Figure 9A). Overall, the number of fruits per plant increased 174% and 62% for these two pepper varieties following exposure to TC09 as compared to tissue culture controls. When fruit weight was determined, cayenne pepper generated 173.1, 34.2, and 55.4 g of collected fruit per plant; and minisweet pepper produced 417.9, 247.8, and 272.0 g per plant for TC09, direct seeding and tissue culture treatments, respectively (Figure 9B). These results indicate that TC09 treatment gave rise to 213% and 54% yield increase over tissue culture control in cayenne and minisweet peppers, respectively. During the period from early 2017 to late 2018, we conducted a total of two experiments with minisweet pepper and three experiments with cayenne pepper. All results were highly consistent (data not shown).

## Discussion

In recent years there have been an increasing number of reports on MVOC-mediated PGP by beneficial and, in some cases, phytopathogenic microorganisms (Ryu et al., 2003; Zou et al., 2010; Minerdi et al., 2011; Paul and Park, 2013; Kai and Piechulla, 2014; Kanchiswamy et al., 2015a; Sánchez-López et al., 2016b; Cordovez et al., 2017; Tahir et al., 2017). Reported MVOCs are able to stimulate photosynthesis, cell expansion and starch accumulation, early flowering and fruit development and enhance host defense and stress tolerance (Ryu et al., 2004; Zhang et al., 2007; Ahemad and Kibret, 2014; Pieterse et al., 2014; Liu and Zhang, 2015; Chung et al., 2016; Sánchez-López et al., 2016a; Cordovez et al., 2017). This work has highlighted potential benefits and opportunities to utilize MVOC-mediated technology to improve agriculture in an environmentally friendly and sustainable fashion (Berg, 2009; Kanchiswamy et al., 2015a; Baez-Rogelio et al., 2016). However, with the relatively low levels of growth enhancement demonstrated thus far and the few plant species studied, the practical application involving direct use of MVOC emitting microorganisms has remained limited (Song and Ryu, 2013; Li et al., 2016; Cordovez et al., 2017; Fincheira and Quiroz, 2018). Furthermore, all effective MVOC species, their interactions and direct correlation between MVOC and plant response need to be determined. In this study, we demonstrated that short term exposure *in vitro* to an environmentally isolated fungal strain TC09 identified as *C. sphaerospermum* dramatically promoted tobacco plant growth via MVOCs. We showed in pilot scale experiments under greenhouse conditions that crop productivity of pepper plants could be significantly enhanced by exposure *in vitro* to TC09 at the seedling stage.

The effects of TC09 exposure on plant growth were largely correlated with the duration of exposure. The stem length of treated tobacco plants doubled after 20 days relative to controls with as little as a 24-h exposure. Exposure duration of 10 days gave maximal PGP as durations beyond 10 days had no significant additive effect. These observed dynamic changes in growth stimulation could potentially be explained by the loss of MVOC emission by TC09 over longer culturing times, saturated plant signaling receptors, or alternatively as a consequence of the depletion of nutrients in the media by rapidly growing plants. To test the latter hypothesis, we performed experiments to test the requirement for sucrose in the tissue culture media and found that sugar concentrations of >5 g/L were necessary for the observed PGP activity but did not enhance PGP beyond 10g/L. These data suggest that endogenous sugar production in young plants is not sufficient to support robust PGP by TC09 and that sustained PGP requires exogenous carbon sources. This finding may also imply that TC09 MVOCs function by activating some signaling pathway(s) in the plant that allows it to better utilize carbon resources available directly through root uptake.

Another contributing factor to PGP by TC09 was the fungal cultivation medium. MS medium supported the highest level of PGP while some common fungal growth media such as Hutner’s medium not only suppressed fungal growth but also reduced PGP activity. MVOC-mediated PGP has been previously shown to vary according to microbial culture conditions. By using rhizobacteria *Bacillus mojavensis* RRC101, Rath et al. (2018) demonstrated such variability in microbial metabolism in conjunction with PGP activity. When cultured on ½ strength MS medium, the bacteria produced known PGP compounds acetoin and 2,3-butanediol, thus leading to improved growth of Arabidopsis seedlings. However, the use of nutrient agar for bacterial cultivation resulted in the production of relatively high levels of acetone and 2-propanone and correlated with phytotoxicity. In addition, the microbial volatile compound, e.g., β-caryophyllene which is commonly produced by fungi was shown to switch its mode of action from stimulatory to inhibitory depending on concentrations (Wang et al., 2009). Other studies also established that both the diversity and absolute quantity of MVOCs emitted by many microbes are dictated by growth conditions and external stimuli (Sunesson et al., 1995; Weikl et al., 2015; Lee et al., 2016; Nieto-Jacobo et al., 2017). Our findings of altered PGP activities derived from the use of different fungal culture media suggest that culture conditions for TC09 and other PGP microbes can be further exploited and optimized to achieve more effective PGP.

The observed levels of PGP upon exposure of tobacco seedlings to TC09 greatly exceeded those previously reported using other microorganisms (Ryu et al., 2003; Minerdi et al., 2011; Paul and Park, 2013; Bitas et al., 2015; Lee et al., 2016; Sánchez-López et al., 2016b; Cordovez et al., 2017; Tahir et al., 2017). We found that varied levels of PGP activity were also exhibited by diverse species of *Cladosporium*, however, this activity was largely strain specific. Paul and Park (2013) previously reported an approximately 10-fold increase in fresh weight of tobacco plants exposed to *C. cladosporioides* CL-1 cultured on PDA for 28 days (4 weeks). The results reported here suggest that differences between various studies could be due to several factors including the specific strain or isolate used, the culture media used for both the plant and fungus, and the availability of sufficient carbon resources to support maximal plant growth rates. Nevertheless, the rapid response to growth stimulants should facilitate further investigation of the mechanisms underlying PGP activity as recently outlined by Fincheira and Quiroz (2018).

A potential limitation for the use of MVOC-mediated PGP is plant damage resulting from the use of phytopathogens or microbes with parasitic behaviors. Phytopathogenic microorganisms can cause adverse effects on target plants in the case of escape and contamination during fungal exposure. In this study, TC09 did not induce any discernable pathogenic responses in all test plants, consistent with its non-pathogenic nature. The non-pathogenic behavior of TC09 may be attributed to its environmental origin and non-adaptive relationship with plants. *C. sphaerospermum* has long been known as one of the most ubiquitous air-borne, saprobic fungal species and can be found in diverse environments, air and physical structures (Park et al., 2004; Zalar et al., 2007). In contrast, we observed leaf tissue necrosis in tobacco treated with *C. sphaerospermum* NRRL 8131 (data not shown). This particular isolate was originally referenced as *Cladosporium lignicolum* Corda based on its sylvan habitat and the ability to degrade and absorb nutrients from lignified woody materials (Dugan et al., 2008). Even though it promoted plant growth similar to TC09, its ability to induce phytotoxic reactions in target plants suggests pathogenic propensity. The ability of TC09 to significantly promote plant growth via MVOCs while lacking harmful phytotoxic effects makes it particularly attractive for further exploitation and the development of practical applications to improve crop productivity.

Transcriptome analyses previously revealed the correlation between differential gene expression and PGP as well as enhanced tolerance to biotic and abiotic stresses following treatment with beneficial microorganisms and MVOCs (e.g., van Loon, 2007; Zhang et al., 2007; Sánchez-López et al., 2016a). Our RNAseq data from untreated and treated tobacco plants provided evidence that diverse metabolic and signaling pathways are altered as a result of exposure to TC09. The most striking observation was that TC09 treatment was accompanied by down-regulation of photosystem functions. This finding is somewhat inconsistent with several previous reports that MVOCs from various microbes caused substantial positive impacts on photosynthesis and chlorophyll production (see review by Fincheira and Quiroz, 2018). The transcriptome data also revealed a potential increase in cell wall metabolism including up-regulation of numerous cellulose synthase genes, endoglucanases, pectinesterases, fasciclins, and xyloglucan endotransglucosylases. A number of putative expansins were also induced which are known to induce slippage of cellulose microfibrils via a non-enzymatic cell wall loosening mechanism and are responsible for cell size increase (Sampedro and Cosgrove, 2005; Choi et al., 2008). In previous MVOC-related studies, *Fusarium oxysporum* reportedly was able to upregulate expression of an expansin A5 gene in lettuce (Minerdi et al., 2011). Volatiles from rhizobacteria *B. subtilis* (GB03) also reportedly increased expression of eight expansin genes ∼2-fold (Zhang et al., 2007). Our results indicate that up to 11 expansin genes in treated tobacco plants were up-regulated twofold to fivefold (five genes with more than threefold) over the control.

The rapid growth enhancement following MVOC or microbial exposure may also reflect promoted mitosis and cell cycle that give rise to increased cell numbers. We identified a number of cyclins and CDKs as DEGs in tobacco plants exposed to TC09. Mitotic-specific cyclins and CDKs are known to interact in partner-dependent fashion to form active complexes that function to modulate cell cycle during different phases of cell division (Mironov et al., 1999). This process is also regulated by mitogenic stimulation through protein docking, CDK phosphorylation, and proteolytic degradation of cell cycle proteins (Breyne et al., 2002). Homeostatic alterations in the expression of these proteins has been linked to either faster or slower cell multiplication (Komaki and Sugimoto, 2012). Noticeably, a previous study reported that ectopic over-expression of a cyclin B in Arabidopsis resulted in promoted cell proliferation in roots (Doerner et al., 1996). Our findings provide a direction for further study to address the influence of MVOCs on cell cycle regulation.

Phytohormones including cytokinin, GA3 and auxin can modulate plant growth and development and their activities are dictated by either biosynthesis or homeostasis of active forms. Interestingly, we observed a reduction (2- to 10-fold) in six UDP-glucose glycosyltransferases (UGGTs), which play an important role in the deactivation, homeostasis, storage and secretion of numerous macromolecules including phytohormones via glycosylation modification (Jones and Vogt, 2001; Ostrowski and Jakubowska, 2014). Kim et al. (2015) also reported the down-regulation of a glycosyltransferase in tobacco in response to MVOCs emitted by rhizobacteria *B. subtilis* although the functional involvement was not elucidated. We postulate that down-regulation of this class of genes may reduce UGGT activity. This consequently leads to minimized deactivation of phytohormones thus potentially increasing the quantity of active phytohormones galvanizing various aspects of plant growth. Early flowering induced by MVOCs was previously attributed to the enhancement of cytokinin action (Sánchez-López et al., 2016b).

Our transcriptome data indicated that the core transcriptional regulator JA-1 in jasmonic acid (JA) signaling pathway was up-regulated more than twofold in tobacco plants exposed to TC09. Also, many herbivore defense genes were up-regulated as well, including proteinase inhibitor II (21-fold), seed storage type protease inhibitor (3.6-fold), Kunitz trypsin inhibitor (2.3-fold) and three cytochrome P450 proteins with sequences homologous to a previously reported protein associated with wound healing and pest resistance (Noordermeer et al., 2001; Dunaevsky et al., 2005). Increased expression levels (threefold to fourfold) of eight latex-like proteins were identified which have been shown to have insecticidal activities against herbivore pests (Konno, 2011). Likewise, seven *O*-methyltransferases belonging to a class of catalytic enzymes involved in biosynthesis pathway of FCs also showed elevated expression (2- to 15-fold) (Bourgaud et al., 2006, 2014). FCs are secondary metabolites that function mainly as insect repellants (Nitao et al., 2003). These findings prompt future studies to evaluate whether TC09-exposed plants exhibit enhanced resistance to insect pests. Indeed, previous studies have likewise identified MVOC-induced expression of genes associated with JA signaling pathway, broad-spectrum induced systemic resistance (ISR) and herbivore resistance (Song et al., 2013; Naznin et al., 2014; Kanchiswamy et al., 2015a; Chung et al., 2016).

In this study, we demonstrated significant stimulation of plant growth, early reproduction and fruit yield increase in pepper by exposing seedlings to TC09 for a relatively short duration. Although results were achieved under greenhouse conditions, the high level of productivity increase without the additional input of other resources warrants further investigation as to whether such technology could be practically applied at a production scale. Many considerations have to be taken into account including microbe cultivation and conditions, MVOC deployment and dosage control, plant treatment stage and duration, post treatment process and transplanting methodology. In addition, future investigations are needed to study the genome, transcriptome and proteome of TC09 to identify genes, pathways and regulatory mechanisms associated with VOC production and PGP activity. The TC09 strain described here provides a useful tool to carry out such studies and highlights the potential of microbe-based PGP technology to benefit agriculture in an environmentally friendly and sustainable fashion.

## Author Contributions

ZTL, WJJ, and CD conceived the concept and designed the experiments. ZTL performed *in vitro* experiments, analyzed the data, and drafted the manuscript. CD carried out transcriptome assembly and analyses. WJJ, WMJ, and BE conducted fungal identification experiments. ZL and AC provided scientific advice and discussed results. ZTL, CD, WJJ, WMJ, ZL, and AC reviewed the final version of the paper.

## Conflict of Interest Statement

The authors declare that the research was conducted in the absence of any commercial or financial relationships that could be construed as a potential conflict of interest.

## Supplementary Material

Figure S1 Neighbor-joining dendrogram of ITS sequences associated with TC09. The 249 bp ITS3/4 amplicon consensus sequence derived from PCR amplification using DNA samples of the fungal isolate was used to search GenBank database for homologous sequences. More than 148 sequence entries were retrieved and subject to phylogenetic analysis. Neighbor-joining method was used to reconstruct the phylogenetic tree with bootstrap values indicated for each branch. Sequence entries belonging to same species were labeled using identical colors. Asterisk indicates the caudal position of the fungal isolate within the monophyletic group *Cladosporium sphaerospermum*.

Figure S2 Heat map depicting high (red) and low (blue) relative levels of differentially expressed genes (DEGs) in individual plants with and without exposure to TC09. Six-day-old tobacco seedlings were maintained under *in vitro* conditions with or without a 10-day exposure to TC09 and then subject to total RNA extraction using shoot tip tissues that include apical meristem and three to four young leaves. Heat map was generated by using CLC Genomics Workbench software program following transcriptome assembly using tomato transcriptome as a reference. Plant treatment type is marked by red (control) and blue (TC09-treated) line on top. There were four replicated individual plants for each treatment. Color-rendered scale of expression changes is marked on bottom.

Figure S3 Colony of *Cladosporium sphaerospermum* TC09 on PDA medium **(A)**; and confocal microscopy micrographs of branching chains of conidia **(B)**; intercalary and terminal conidia **(C)**; and mycelium **(D)**.

Table S1 List of all tobacco DEGs after exposure *in vitro* to TC09 for 10 days. Fold change in expression levels was calculated based on means of four biological replicates for both control and MVOC-treated plants. Genes were annotated using GO terms and tomato (*Solanum lycopersicum*) reference genes available on https://solgenomics.net.
