# Supplementary figures and images for: Exposure in vitro to an Environmentally Isolated Strain TC09 of Cladosporium sphaerospermum Triggers Plant Growth Promotion, Early Flowering, and Fruit Yield Increase
Source: Front Plant Sci. 2019 Feb 1;9:1959. doi: 10.3389/fpls.2018.01959 (PMC6367233; doi:10.3389/fpls.2018.01959)

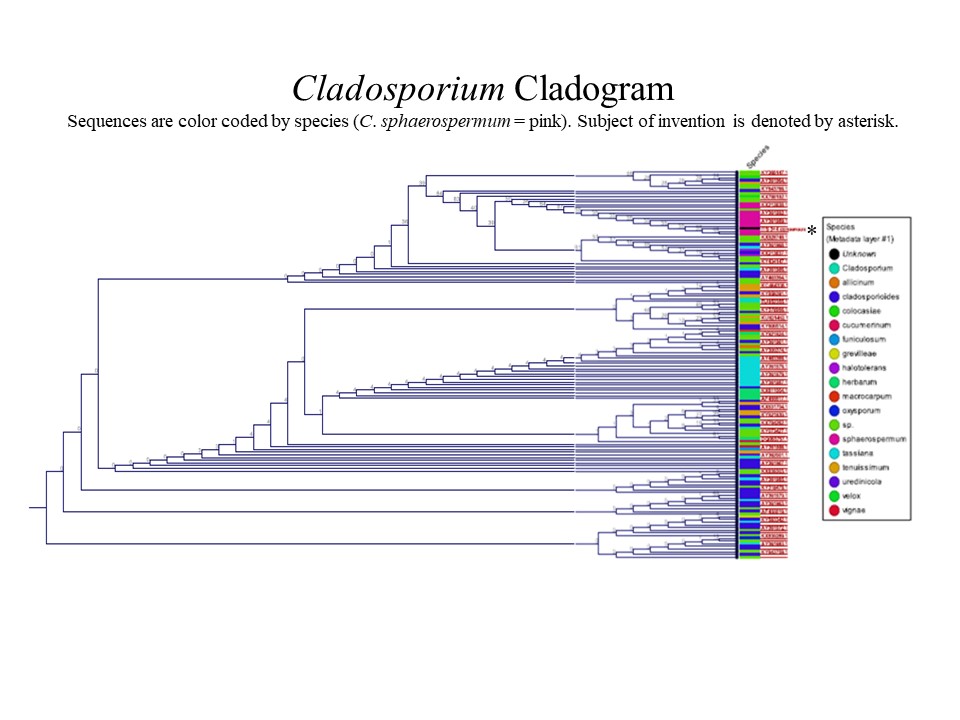

Supplement: Figure S1 — Neighbor-joining dendrogram of ITS sequences associated with TC09. The 249 bp ITS3/4 amplicon consensus sequence derived from PCR amplification using DNA samples of the fungal isolate was used to search GenBank database for homologous sequences. More than 148 sequence entries were retrieved and subject to phylogenetic analysis. Neighbor-joining method was used to reconstruct the phylogenetic tree with bootstrap values indicated for each branch. Sequence entries belonging to same species were labeled using identical colors. Asterisk indicates the caudal position of the fungal isolate within the monophyletic group Cladosporium sphaerospermum. [file Image_1.JPEG]

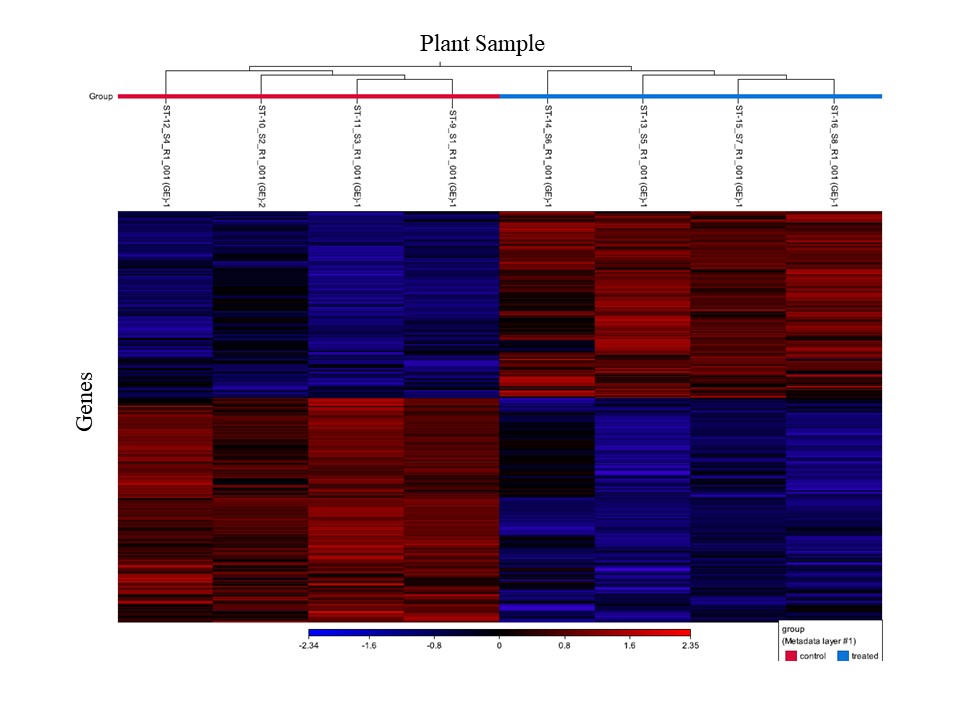

Supplement: Figure S2 — Heat map depicting high (red) and low (blue) relative levels of differentially expressed genes (DEGs) in individual plants with and without exposure to TC09. Six-day-old tobacco seedlings were maintained under in vitro conditions with or without a 10-day exposure to TC09 and then subject to total RNA extraction using shoot tip tissues that include apical meristem and three to four young leaves. Heat map was generated by using CLC Genomics Workbench software program following transcriptome assembly using tomato transcriptome as a reference. Plant treatment type is marked by red (control) and blue (TC09-treated) line on top. There were four replicated individual plants for each treatment. Color-rendered scale of expression changes is marked on bottom. [file Image_2.JPEG]

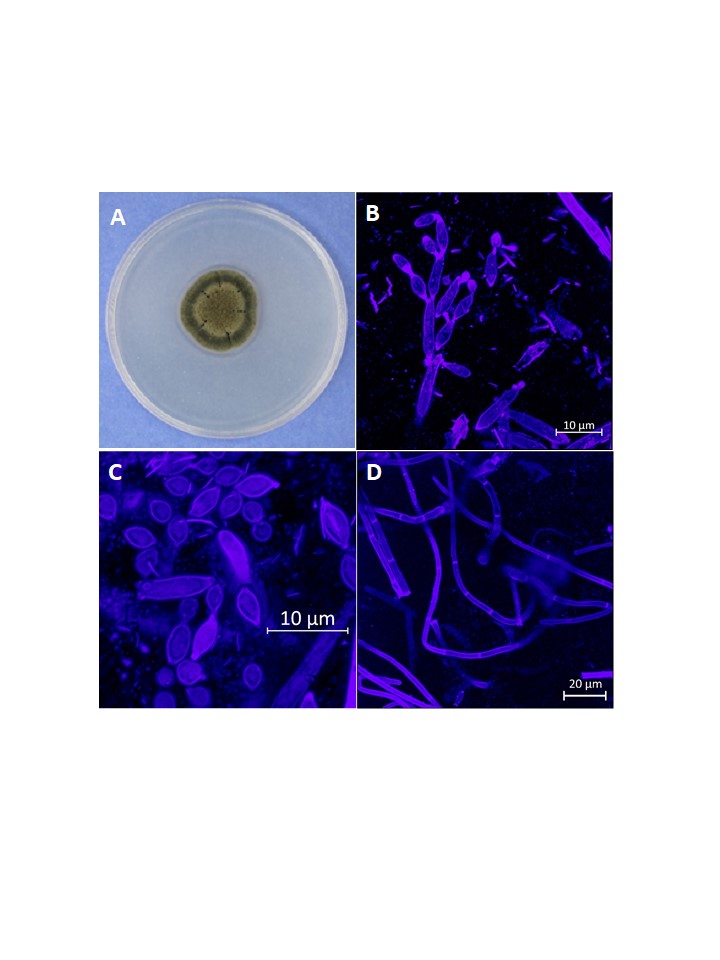

Supplement: Figure S3 — Colony of Cladosporium sphaerospermum TC09 on PDA medium (A); and confocal microscopy micrographs of branching chains of conidia (B); intercalary and terminal conidia (C); and mycelium (D). [file Image_3.JPEG]
